# Challenges and Perspectives for Prevention of Infectious Diseases

**DOI:** 10.3390/vaccines9060571

**Published:** 2021-06-01

**Authors:** Yutaka Ueda

**Affiliations:** Department of Obstetrics and Gynecology, Graduate School of Medicine, Osaka University, Osaka 565-0871, Japan; y.ueda@gyne.med.osaka-u.ac.jp

The world is currently engaged in an ongoing battle against COVID-19. One aim is the development of a vaccine and the search for an effective treatment. This is a purely medical effort. The other is to promote preventive behavior among people. This can be called behavioral science. The WHO has identified vaccine hesitancy as one of the 10 threats to global health. One of the biggest causes of vaccine hesitancy is an inaccurate understanding of infectious diseases and vaccines. In this special edition of “Human Consciousness and Behavior towards Infectious Diseases and Vaccines”, important studies on people’s knowledge and attitudes toward infectious diseases and their prevention, as well as research that contributes to the effective dissemination of vaccines, will be reported.

First, Dr. Azizul Haque et al. [1] discusses the advantages and disadvantages of various vaccine platforms and evaluates the safety and efficacy of vaccines. Once a vaccine is developed, the next challenge will be acquisition, deployment, and uptake. The present manuscript describes these challenges in detail and proposes solutions to the vast array of translational challenges.

Next, Dr. Dimitrios Papagiannis et al. [2] evaluates the acceptability of vaccination against COVID-19 among health professionals two weeks prior to the start of the Greek vaccination campaign against COVID-19. A cross-sectional online survey demonstrated a high level of acceptance for the COVID-19 vaccine (78.5%) and a high vaccination coverage for the influenza vaccine. Age > 45 years, absence of fear over vaccine safety, and information received from the Greek public health authorities were found to be independent factors associated with the likelihood of COVID-19 vaccination acceptance.

Dr. Arriel Benis et al. [3] present clear evidence that, contrary to the prevailing public perceptions, young audiences using social media have mostly a positive attitude towards COVID-19 vaccination. Another important finding was the younger population’s fear of personal COVID-19 infection. These results enable a practical public-messaging pathway to reinforce vaccination campaigns addressing the younger population.

Dr. Minyi Zhang [4] investigated the willingness to accept the 23-valent pneumococcal polysaccharide vaccine (PPSV23) and influencing factors among people aged over 60 years during the COVID-19 pandemic. In China, where the pneumococcal vaccine coverage presents extremely low among elderly people, 91.5% presented a positive attitude toward PPSV23. Logistic analyses suggested the influencing factors included knowledge about pneumonia, perception of the seriousness of pneumonia and preventative methods for pneumonia, worries about getting pneumonia, understanding vaccine policy, and influenza vaccine and PPSV23 histories. 

On the other hand, there is a vaccine for which vaccination has completely stagnated, even in such a coronary pandemic. The WHO has listed HPV as one of the major threats to health, but the vaccination rate has completely stopped at less than 1%, and it is certain that cervical cancer will occur more frequently in Japan than anywhere else in the world. 

In this context, Dr. Risa Kudo R et al. [5] conducted the first large-scale survey analyzing the association between HPV vaccination and sexual activity in Japanese girls. Not only do unvaccinated girls not benefit from vaccines, but they also tend to engage in high-risk sexual behavior, and thus it was shown to be even more important to provide information on the effectiveness of vaccines and the usefulness of cancer screening.

Dr. Asami Yagi et al. [6] analyzed the results of an Internet survey of mothers of HPV vaccine recipients over time. Mothers’ intentions to get their daughters HPV vaccinated is still quite low, and vaccination rate would not easily recover by providing information on the effectiveness and safety of HPV vaccine.

In Japan, the HPV vaccination rate was as high as around 70% in the generation born between 1994 and 1999. These women are now beginning to be included in Japan’s cervical cancer screening program, which targets women aged 20 and older. Dr. Mariko Taniguchi et al. [7] investigated the health attitudes of vaccinated and non-vaccinated women of the HPV vaccination generation, and developed a method for recommending cervical cancer screening according to HPV vaccination status. This is important data for the HPV vaccine cessation generation as they become eligible for cervical cancer screening in the near future.

We hope that this special edition of “Human Consciousness and Behavior towards Infectious Diseases and Vaccines” will contribute to the improvement in health of people around the world.

## Data Availability

The data that support the findings of this study are available from the corresponding author, upon reasonable request.
